# Experiencias interactivas y conversacionales de familias visitantes en “Darwin, la exposición,explorando las especies”, en el Museo Trompo Mágico, México

**DOI:** 10.1590/S0104-59702023000100051

**Published:** 2023-10-23

**Authors:** Luisa Massarani, Ana Claudia Nepote, Jessica B. Carneiro, Bruna Ibanes Aguiar, Graziele Scalfi

**Affiliations:** i Instituto Nacional de Comunicação Pública da Ciência e Tecnologia Casa de Oswaldo Cruz Fiocruz Rio de Janeiro RJ Brasil luisa.massarani@fiocruz.br Coordinadora, Instituto Nacional de Comunicação Pública da Ciência e Tecnologia; Investigadora, Casa de Oswaldo Cruz/Fiocruz. Rio de Janeiro – RJ – Brasil luisa.massarani@fiocruz.br; ii Escola Nacional de Estudos Superiores Universidad Nacional Autónoma do México Morelia MICH México nepote@enesmorelia.unam.mx Profesora asociada, Escola Nacional de Estudos Superiores/Universidad Nacional Autónoma do México. Morelia – MICH – México nepote@enesmorelia.unam.mx; iii Instituto Nacional de Comunicação Pública da Ciência e Tecnologia Casa de Oswaldo Cruz Fiocruz Rio de Janeiro RJ Brasil jessicabcarneiro@gmail.com Investigadora, Instituto Nacional de Comunicação Pública da Ciência e Tecnologia/Casa de Oswaldo Cruz/Fiocruz. Rio de Janeiro – RJ – Brasil jessicabcarneiro@gmail.com; iv Instituto Nacional de Comunicação Pública da Ciência e Tecnologia Casa de Oswaldo Cruz Fiocruz Rio de Janeiro RJ Brasil brunaibanes@yahoo.com.br Investigadora, Instituto Nacional de Comunicação Pública da Ciência e Tecnologia/Casa de Oswaldo Cruz/Fiocruz. Rio de Janeiro – RJ – Brasil brunaibanes@yahoo.com.br; v Instituto Nacional de Comunicação Pública da Ciência e Tecnologia Casa de Oswaldo Cruz Fiocruz Rio de Janeiro RJ Brasil graziscalfi@gmail.com Investigadora, Instituto Nacional de Comunicação Pública da Ciência e Tecnologia/Casa de Oswaldo Cruz/Fiocruz. Rio de Janeiro – RJ – Brasil graziscalfi@gmail.com

**Keywords:** Familias, Educación informal, Aprendizaje de libre elección, Families, Informal education, Free choice learning

## Abstract

Analizamos el contenido conversacional y las interacciones de diez familias, con el fin de comprender cómo es la experiencia de aprendizaje en una exposición científica. Como herramienta de análisis se utilizó un protocolo que combina aspectos teóricos y empíricos de la interactividad. Los resultados evidencian que las familias participaron activamente en la exhibición, observando y conversando sobre los animales, haciendo preguntas, buscando respuestas y elaborando explicaciones basadas en el pensamiento científico. Los adultos actuaron como facilitadores del aprendizaje y para ello se apoyaron en paneles informativos fomentando la conexión con experiencias previas. Los niños muestran curiosidad, emociones y comportamientos que evidencian sus experiencias de aprendizaje e interés por temas científicos.

## Abstract (en)

We analyzed the conversational content and interactions of ten families, with the aim of understanding the learning experience of families in a scientific exhibition. As instrument of analysis, it was used a protocol combining theoretical and empirical aspects of interactivity. The results show that the families actively participated in the exhibition, observing and talking about the animals, asking questions, looking for answers and elaborating explanations based on scientific thinking. The adults acted as facilitators of learning and for this they were supported by information panels promoting the connection with previous experiences. Children show curiosity, emotions and behaviors that evidence their learning experiences and interest in scientific topics.

La relación entre los museos de ciencia y la sociedad ha cambiado significativamente a lo largo del tiempo en respuesta a su pluralidad y a los avances en las concepciones sobre el papel de los museos en el aprendizaje (Gruzman, Siqueira, 2007; Souza, Cunha, 2020). McManus (1992) clasificó los museos en tres generaciones, a saber: (1) historia natural: tenían una estrecha relación con la academia y comunicaban la investigación científica con el público utilizando un lenguaje académico y autoritario, a través de una exhibición de objetos tradicionales; (2) ciencia e industria: estaban más cerca del público, sus exposiciones eran más atractivas y estimulantes y estaban orientadas a la educación (masiva) del ciudadano para satisfacer las necesidades de la industria; y (3) fenómenos y conocimientos científicos: también están cerca al público, pero se preocupan por la enseñanza y su énfasis está en la ciencia y la tecnología.

La estructura cronológica proporcionada por McManus (1992) es importante para darnos un sustento de la historia de la ciencia involucrada en estos espacios. Sin embargo, como argumenta Friedman (2010), las características de estos museos a lo largo del tiempo se superpusieron y también sus objetivos principales, como la conservación, la investigación, la formación y la recolección. La educación, a su vez, se explora más con los museos de tercera generación.

En este sentido, algunos autores (por ejemplo, Cazelli et al., 2002) se refieren a una nueva generación de museos y a una pedagogía museal con experiencias de aprendizaje intencionales y adecuadas para que las exposiciones estimulen la interacción entre los visitantes, a través de la aproximación de ciencia a su vida diaria, destacando su importancia para la transformación social.

Así, los museos de ciencia contemporánea se caracterizan por exhibiciones interactivas compuestas por objetos de naturaleza semiótica y variada que tienen como objetivo transformar la forma en que los visitantes se relacionan con la exposición (Davallon, 2010), por lo tanto, la selección de objetos que la integrarán debe ser llevada a cabo dentro de una perspectiva pedagógica bien definida por parte del museo (Pol, Noguera, Asensio, 2016). En esta perspectiva, los objetos se destacan por la forma en que atraen y estimulan al visitante ya que permiten la conexión entre ideas y fenómenos científicos abstractos. Achiam, May y Marandino (2014) los discuten utilizando el concepto de *affordance*, que trata de las características que permiten su comprensión sin necesidad de explicación y fomenta el uso crítico de estos objetos por parte de los diseñadores para crear un entorno que permita al museo comunicarse con una audiencia diversa, especialmente cuando se trata de una exposición no guiada.

Falk y Dierking (2000, 2014) señalan que al interactuar con la exposición y entre ellos, los visitantes se encuentran con lo físico (arquitectura, disposición de módulos y objetos), lo sociocultural (experiencias compartidas con quienes viven o con quienes realizan la visita), y lo personal (expectativas, motivaciones, conocimientos e intereses previos); y que es en la intersección de estos contextos donde se vive la experiencia interactiva. Bajo esta interpretación, el aprendizaje se entiende como un esfuerzo contextual para la construcción de significados que se da en el tiempo y permea los tres contextos mencionados. Además, se reconoce como de libre elección, ya que los visitantes tienen autonomía para controlar y elegir qué, cuándo y cómo aprenderán (Falk, Storksdieck, 2005).

Según Furman et al. (2018), se espera que el aprendizaje permita al sujeto desarrollar negociaciones con sus pares a través de altos niveles cognitivos, lo que implica utilizar la capacidad crítica y creativa para resolver problemas complejos, analizar y posicionarse a través de los desafíos vividos en el día a día. Aunque este es uno de los objetivos escolares deseables para el siglo XXI (Binkley et al., 2012), es importante señalar que el aprendizaje no se limita a entornos formales (Falk, Dierking, 2010) y comienza con la familia (Dierking, 2005).

Un factor que vale la pena mencionar es que las familias representan la mayor audiencia entre todos los visitantes de los museos de ciencia, especialmente en América del Norte y Europa (ASTC, 2016; SMG, 2018), este hecho contribuye a que la mayoría de los estudios disponibles sobre el aprendizaje en los museos son de estas regiones. Si bien estos resultados ayudan a investigadores de otras regiones a diseñar los estudios: los contextos en los que se produce el aprendizaje son diferentes dada la estrecha relación entre museo-aprendizaje-cultura familiar. En el contexto latinoamericano este tipo de estudio es poco común (Briseño-Garzón, 2010), aunque se trata de una audiencia recurrente en los museos de ciencia de la mayoría de los países de la región, como el Trompo Mágico (Amieva et al., 2011), el lugar de este estudio.

En este sentido el objetivo de este estudio fue analizar las interacciones y el contenido conversacional de las familias durante una visita a una exposición ubicada en el Museo Trompo Mágico (Guadalajara, México) para comprender las experiencias de aprendizaje y el interés por temas científicos.

## Aprendizaje de libre elección en el museo de ciencias

Al estudiar las experiencias de aprendizaje familiar en los museos de ciencia nos apoyamos en un concepto de aprendizaje alineado con la perspectiva sociocultural que comprende el desarrollo cognitivo basado en la interacción social mediada por actividades contextualizadas (Rowe, O’Brien, 2016). Así, se consideran interacciones en el momento de la visita, conexión con conocimientos previos, con experiencias vividas y emociones que surgen de este proceso. Para ello, analizamos el habla y los gestos (individuales y colectivos) para comprender cómo una exposición se conecta con el contexto personal y conduce a un aprendizaje más profundo, permitiendo que los individuos asignen significado a las experiencias que tienen (Falk, Dierking, 2014; Massarani et al., 2019a).

Cuando las familias visitan museos es necesario tener en cuenta que cada uno de ellos tiene diferentes objetivos, que van desde un momento de ocio hasta el aprendizaje (Callanan, 2012). Es a raíz de estos múltiples objetivos que las familias elegirán lo que visitarán y en la medida en la que se involucrarán con las exposiciones (Ellenbogen, 2002). Ash (2003), a través de una investigación dialógica con familias, mostró que el contenido conversacional es un indicador de aprendizaje. Callanan et al. (2017), Ellenbogen (2002) y Guimarães et al. (2019) señalan conductas que indican aprendizaje, pero enfatizan que puede llegar a niveles superficiales o profundos, dependiendo de las interacciones entre los miembros de la familia, especialmente si todos se comprometen a compartir puntos de vista para la construcción conjunta del conocimiento y la apropiación de los temas expuestos.

En este sentido, Borun, Chambers y Cleghorn (1996) muestran el papel de los adultos como decodificadores y contextualizadores de información y conceptos científicos para hacerlos más comprensibles y relacionarlos con experiencias vividas. En general, esta facilitación se da a través de la acción colaborativa con niños y adolescentes en situaciones que las familias consideran atractivas para ellos y favorables al aprendizaje. Según Crowley et al. (2001), Callanan et al. (2017) y Vandermaas-Peeler, Massey, Kendall (2015), cuando son apoyados por adultos, niños y adolescentes entablan conversaciones explicativas, prueban hipótesis y extraen conclusiones, esto da como resultado un mayor interés e implicación con la exposición y explora los conceptos más en profundidad, factores que facilitan el aprendizaje de conceptos complejos en el futuro.

Si bien los adultos no siempre dominan los conceptos científicos, cabe señalar que tienen la capacidad de involucrar a sus hijos con el tema abordado por la exposición a través de conexiones con experiencias previas (Borun, Chambers, Cleghorn, 1996). Al considerar el papel de decodificador que asumen los adultos y el de aprendiz que asumen los niños y adolescentes, Gaskins (2016) mostró que familias de diferentes culturas (europeo-americano, afroamericano y latino) que visitan el Chicago Children’s Museum (Museo de los niños de Chicago – Chicago, EEUU) tienen dinámicas igualmente diferentes para explorar la exposición, interactuar y concebir el aprendizaje. Este resultado refuerza la importancia de comprender el contexto sociocultural de las familias para el estudio del aprendizaje en los museos.

## El alcance del estudio

En este estudio con enfoque cuantitativo y cualitativo, analizamos las interacciones de las familias en visitas espontáneas a la exposición temporal “Darwin, la exposición, explorando las especies”, reunida en el Museo Trompo Mágico, en Guadalajara, México. Se caracteriza por ser un estudio de caso, cuya investigación empírica se realizó sobre un fenómeno contemporáneo dentro de su contexto, ya que los límites entre el fenómeno y el contexto no están claramente definidos (Yin, 2001). El estudio forma parte de un proyecto más amplio desarrollado en el ámbito del Instituto Nacional de Comunicación Pública de la Ciencia y la Tecnología de Brasil, con el objetivo de comprender los aprendizajes en los museos de ciencia y los significados creados por los diferentes públicos en torno a las actividades de educación no formal que se ofrecen en estos espacios científico-culturales (Massarani et al., 2019a, 2019b, 2019c).

## El lugar y la exposición de estudio

El Museo Trompo Mágico es una institución pública que depende del Gobierno del Estado de Jalisco (México) y se ubica en el municipio de Zapopan, dentro del Área Metropolitana de Guadalajara. Como ocurre en gran parte de México, los contrastes socioeconómicos son profundos. A unos cuantos metros del Museo se encuentra una zona de muy alta plusvalía residencial, mientras que a un kilómetro de distancia se ubica una de las zonas más marginadas del municipio de Zapopan. No necesariamente los vecinos con alta plusvalía son los visitantes comunes del museo (México, 2009).

El museo fue fundado el 30 de abril de 2003, sus construccion y operacion son financiadas totalmente por el Gobierno del Estado de Jalisco. El público objetivo del museo son niños y jóvenes. Aproximadamente un 70% de los visitantes al museo es público proveniente de educación básica. Aunque también reciben visitantes de otros niveles educativos y socioeconómicos y sobre todo familias (México, 2009). Según Orozco Gómez (2004), el museo se reconoce a sí mismo como uno de “cuarta generación” y actúa intencionalmente como un mediador pedagógico comprometido con sus visitantes para involucrarlos física, emocional y mentalmente con sus contenidos, asignándoles roles activos en el aprendizaje.

El museo opera de martes a domingo, y el costo general de ingreso es de cuarenta pesos por persona (aproximadamente dos dólares americanos), equivalente a menos de la mitad del costo de un boleto de cine. Los jueves, la entrada es libre de costo. Existe también un programa de apoyo a adultos de la tercera edad para que ingresen al museo sin necesidad de pagar. Si la visita se realiza como parte de un grupo escolar organizado, el ingreso cuesta veinte pesos mexicanos (aproximadamente un dólar americano) por persona, esta es la misma tarifa de ingreso para estudiantes o profesores con credencial vigente (México, 2009).

La exposición estudiada es la más grande entre las exposiciones temporales que ha producido el Museo Americano de Historia Natural en Nueva York, que visitó 13 países y llegó a cuatro millones de visitantes. Para su exhibición, el equipo educativo del museo realizó una interpretación y adaptación de los contenidos al contexto mexicano y particularmente a Jalisco. Las cuatrocientas piezas que conforman la exposición se distribuyeron en un área de 1.250 metros cuadrados para representar la vida y obra de Charles Darwin (1809-1882) en el Museo Trompo Mágico de Jalisco (México, 29 oct. 2015).

Se organizaron diez módulos expositivos en una lógica lineal y secuencial para que los visitantes pudieran recorrerlos de forma autónoma. Los módulos cubrieron los temas: (1) El mundo antes de Darwin; (2) El joven naturalista; (3) Un viaje alrededor del mundo; (4) Grandes descubrimientos; (5) Una idea toma forma; (6) El trabajo de toda una vida; (7) Estudio de Darwin; (8) La evolución de hoy; (9) Infinidad de formas; (10) Jardín Botánico. En resumen, se retrató el viaje de Darwin alrededor del mundo a bordo del “Beagle”, los descubrimientos que culminaron en la teoría de la evolución por selección natural y los desafíos personales que enfrentó el naturalista hasta la publicación de su teoría. Desde un punto de vista personal, se retrataron sus complejas relaciones familiares y su forma de vida, permitiendo a los visitantes sumergirse en la cultura inglesa en la que él y su familia vivían (Ceci, 2009).

Como objetos destacados, la exposición incluyó réplicas y originales de fósiles, animales vivos y taxidermizados, esqueletos, documentos y plantas de herbario. Además de una gran cantidad de información en paneles para lectura, se diseñaron videos para la contemplación de los visitantes.

## Procedimentos de recopilación de datos

La colecta de datos se realizó en enero de 2016. Al ingresar al Museo Trompo Mágico una integrante del equipo de investigación se acercó a las familias, quien les explicó el propósito del estudio y consultó su disponibilidad para participar en el estudio. Una vez que aceptaron participar, uno de los jefes de familia llenó y firmó el formulario de consentimiento libre e informado; el estudio fue aprobado por el comité de ética de la Fundación Oswaldo Cruz (Caae 10663419.0.0000.5241).

Para registrar las interacciones se eligió a un niño o adolescente integrante de la familia para que portara la cámara subjetiva de GoPro Hero en su cabeza durante el recorrido por la exposición. El punto de encuentro para el inicio de la visita fue cerca de la entrada a la exposición, el investigador encendió la cámara y el grupo inició la visita. La cámara grabó los datos audiovisuales de toda la familia considerando la perspectiva del visitante que la portaba, siguiendo el método de “cámara de punto de vista”, utilizado en estudios en el campo de las ciencias sociales (Glăveanu, Lahlou, 2012) y en estudios previos de este grupo de investigación (Massarani et al., 2019a, 2019b, 2019c, 2019d).

## Características de las familias estudiadas

En nuestro estudio, definimos el número de diez familias participantes con base en la saturación teórica, es decir, la inclusión de nuevas familias no conduce a nuevos elementos y la información se vuelve redundante (Saumure, Given, 2008). Los diez grupos familiares (G1-10) fueron compuestos por 42 sujetos, siendo 27 mujeres (11 adultos, 15 niños y 1 adolescente) y 15 hombres (5 adultos, 7 niños y 3 adolescentes), cuyo número de integrantes, edad grupo, género por grupo familiar y duración de la visita se muestran en la Tabla 1. Definimos grupos familiares como familias compuestas por miembros relacionados biológicamente, o consideradas como miembros de la familia por el grupo en estudio (Briseño-Garzón, Anderson, 2012).

**Tabla 1:** : Información de los miembros de los visitantes de cada grupo familiar (G) y duración de la visita

Grupos	Integrantes	Género/edades	Duración de la visita
G1	4	3♀ (4,8,31); 1♂ (32)	19min17seg
G2	3	2♀ (2,10); 1♂ (21)	22min38seg
G3	3	2♀ (10,39); 1♂ (13)	1h37seg
G4	3	2♀ (8,22); 1♂ (32)	36min45seg
G5	5	4♀ (5,8,20,38); 1♂ (4)	12min45seg
G6	3	2♀ (8,43); 1♂ (12)	1h53seg
G7	5	4♀ (9,9,9,35); 1♂ (44)	1h18min23seg
G8	6	3♀ (4,30,33); 3♂ (3,4,10)	19min56seg
G9	6	3♀(6,10,62); 3♂ (6,13,35)	33min4seg
G10	4	2♀ (14,34); 2♂ (2,8)	20min28seg
Total	**42**	**27♀; 15♂**	**6h4min46seg**

Fuente: Elaborada por los autores.

Con el fin de potenciar la captura de diálogos y acciones a través de la cámara definimos grupos familiares formados por hasta seis sujetos, conteniendo al menos un adulto, un niño o adolescente. Consideramos adultos a personas con al menos 18 años de edad. La delimitación del grupo de edad del niño y adolescente se realizó con base en la legislación del Instituto Mexicano de la Juventud publicada en el Diario Oficial de la Federación en 1999 que considera niño a la persona hasta los 11 años cumplidos y jóvenes a toda persona cuya edad comprende entre los 12 y 29 años de edad (DOF, 6 ene. 1999). Elegimos los grupos de edad de niños y adolescentes porque constituyen una audiencia en edad escolar donde el lenguaje se vuelve más consciente e intencional y se mejoran las operaciones mentales (Elkonin, 1969).

## Protocolo para el análisis de interaciones verbales y no verbales

Los datos fueron analizados utilizando el protocolo desarrollado por los investigadores involucrados en el proyecto que integra este estudio y que, a su vez, fue adaptado de Allard, Boucher (1998), permitiendo investigar la relación entre los tres actores fundamentales de un museo: (1) los módulos expositivos; (2) los mediadores; y (3) los visitantes, en un modelo conocido como el “triángulo pedagógico”. El protocolo de análisis y codificación se divide en diferentes categorías que cubren cinco dimensiones: (1) tipos de interacción; (2) conversación; (3) fotos y videos; (4) cambio; y (5) emoción. En este estudio, adaptamos el protocolo, utilizando las categorías de las dimensiones más expresivas – tipos de interacción, conversación y emoción (Tabla 2). El protocolo utilizado fue validado por seis investigadores y aplicado hasta el momento en 13 museos de ciencia interactivos de América Latina, resultando útil para el área de estudio público (Massarani et al., 2019a, 2019b, 2019c, 2019d).

**Tabla 2:** : Dimensiones de las categorías estudiadas

1. Tipos de interacción	
**1.1. Visitante-visitante**	
	Cuando los visitantes hablan entre ellos, independientemente del contenido de esa conversación, puede ser sobre temas de exhibición y temas que se abordan o no
**1.2. Visitante-módulo expositivo**	
1.2.1. Interacción contemplativa	Contemplación, observación, visualización sin tocar/manipular un módulo expositivo o una parte concreta de este
1.2.2. Lectura de panel/texto/foto	La interacción se da a través de la lectura en voz alta de textos (íntegros o parciales) de paneles informativos, panel, pie de foto y texto de los módulos expositivos
**2. Conversaciones**	
**2.1. Contenido de las conversaciones**	
2.1.1. Conversaciones sobre temas científicos	Diálogos sobre un tema científico, discutir dilemas éticos y morales de la ciencia, el impacto social de la actividad científica, aportar datos o contenido científico etc.
2.1.2. Conversaciones sobre exposición (funcionamiento, diseño, experiencia en el museo)	Diálogo desencadenado por la interacción de los visitantes con la exposición y/o los módulos expositivos, ya sea sobre su funcionamiento, diseño y/o experiencia museística
2.1.3. Conversaciones en las que se hace asociación con experiencias previas y vivencias personales	Movilización, utilización, cuestionamiento sobre los propios conocimientos, creencias, rituales, formas de vida, en la experiencia museal, haciendo referencia a las vivencias de la infancia, conocimientos de la escuela; referencias a películas, libros, series y programas de televisión etc.
**3. Emoción**	
	Expresión de algún sentimiento, positivo o negativo, durante la visita

Fuente: Adaptado de Massarani et al. (2019a).

Para analizar los resultados se utilizó el *software* Dedoose 8.0.23, que categoriza los segmentos de audio y video del corpus, acciones textuales y actitudinales de los visitantes de manera simultánea. Enfatizamos que los códigos pueden superponerse, ya que las categorías del protocolo de análisis no son exclusivas. Para preservar la identidad de los sujetos, los clasificamos como “V” (visitante), siendo el individuo número 1 siempre el niño o adolescente que portaba la cámara. Los datos de estas interacciones se analizaron cuantitativamente (duración de las visitas, número de frecuencia de los códigos y su duración) y cualitativamente (contenido de las conversaciones e interacciones).

## Características de la visita realizada por las familias

A partir del análisis de los videos observamos que las familias G1, G2 y G9 se separaron cuando los miembros se interesaron en diferentes experimentos, pero luego se juntaron para compartir lo que habían visto. En los otros grupos, las familias permanecieron juntas durante la mayor parte de la visita y, en general, V1 pasó por la exposición y fue seguido por los familiares, o en caso contrario, siguió al resto de la familia. Los grupos G3, G6 y G7 se dedicaron mucho a la lectura (juntos, en voz alta y con la mirada fija), a la interpretación de los objetos expuestos y a las conversaciones sobre información y conceptos científicos, siendo estos los grupos que las visitas tuvieron más tiempo: 1h37seg; 1h53seg y 1h18min23seg; respectivamente.

Las interacciones de las familias durante el recorrido por la exposición representaron 6h4min46seg (365 min) de videos analizados. En este momento, se identificaron secciones de cada actividad y se contaron 1.869 aplicaciones de códigos. El tiempo de visita de cada familia osciló entre 12min45seg en G5 y 1h18min23seg en G7, con una media de 36 min. Los extractos referentes a las dimensiones, categoría y subcategorías, número total y tiempo de ocurrencia, así como su relación con el tiempo total de visitas (expresado en porcentaje) se muestran en la Tabla 3.

**Tabla 3:** : Categoría de interacciones, ocurrencia, tiempo de ocurrencia y porcentaje (%) en relación al tiempo total de la visita

Categorías/subcategorias	Ocurrencia	Tiempo de ocurrencia	% tiempo total
**1. Tipos de Interacción**			
1.1. Visitante-visitante	252	264min	72
1.2. Visitante-módulo expositivo			
1.2.1. Interacción contemplativa	292	308min	84
1.2.2. Lectura de panel/texto/foto	248	116min	32
**2. Conversación**			
2.1. Contenido de conversaciones			
2.1.1. Conversaciones sobre temas de ciência	307	155min	42
2.1.2. Conversaciones sobre exposición (funcionamento, diseño, experiencia museal)	339	64min	17
2.1.3. Conversaciones en las que ocurren asociaciones con experiencias previas y vivencias personales	44	11min	3
**3. Emoción**	199	17 min	5

Fuente: Elaborada por los autores.

## ¿Cómo interactuaron las familias durante la visita?

En general las familias interactuaron entre sí a lo largo de la exposición, prueba de ello es que la categoría visitante-visitante (n = 252) correspondió al 72% del tiempo total de la visita. Los episodios que hacen referencia a este código se muestran en los ejemplos 1 a 3.

Ejemplo 1 (G7): V1: Mira papá, el escarabajo tira agua y por culpa del escarabajo [Darwin] se quemó la lengua [Explica después de leer el panel]./ V3: ¿Cómo es eso? [muestra la figura del escarabajo y un chorro de agua]./ V1: Papá, pero ¿estos los hicieron o los atraparon?/ V5: Atrapó y estudió y…/ V1: ¿Pero son de verdad?/ V5: Eso es.

Ejemplo 2 (G4): V1: Mami, un dinosaurio./ V2: No, es una ballena, la mano de la ballena [refiriéndose a la aleta pectoral].

Ejemplo 3 (G1): V1: ¡Mira, la tortuga! ¿Qué es eso?/ V3: Es una libélula en una lupa./ V1: Puaj, qué fea está./ V3: Pero mira obsérvala de este lado que no hay lupa [habla entre risas]./ V1: Bonita./ V3: Y de este otro lado de la lupa que hay?/ V1: Fea. Bonita, Fea./ V4: Mira qué es eso./ V1: Una libélula, está muy fea, parece una mosca, pero con alas grandes.

Los ejemplos muestran que las interacciones entre las familias abarcaron el contenido expuesto y entre las conversaciones observadas destacamos aquellas en las que el visitante señala algo para ser observado por toda la familia, así como conversaciones posteriores a la lectura de paneles (ejemplo 1). Las interacciones también se asociaron con el comportamiento de niños y adolescentes cuando sintieron curiosidad por un determinado objeto (ejemplos 2 y 3).

En algunos casos los adultos participaron más en las discusiones con niños y adolescentes (ejemplos 1 y 3). En el ejemplo 2, la participación del adulto fue en forma de aclarar la duda del niño y, aunque hay un error conceptual en su respuesta, es claro que la estrategia utilizada es acercar el objeto de la curiosidad a la realidad del niño. Como se mencionó anteriormente, en las visitas a las familias, es común que los adultos asuman un rol de liderazgo y guía en situaciones de aprendizaje (Crowley et al., 2001), sin embargo, es importante enfatizar que en todos los ejemplos las interacciones podrían haber generado conversaciones más profundas sobre temas relacionados con la ciencia.

De acuerdo con Callanan et al. (2020), Cerqueira et al. (2016) y Fender y Crowley (2007), el comportamiento de los adultos en relación a los niños y adolescentes en las visitas familiares implica una mayor o menor posibilidad de explorar la exposición y entablar discusiones y reflexiones más profundas sobre el tema expuesto. Según lo observado por nosotros, los niños y adolescentes interactuaban en mayor o menor medida según la receptividad y orientación de los adultos. Para Faria y Chagas (2012) este rol que asume el adulto y, en el caso de su estudio, el docente varía según su interés personal por la exposición e influye en el involucramiento de los niños que se reflejan en la figura adulta y en sus actitudes para desarrollar su propio interés por la exposición.

Vandermaas-Peeler, Massey y Kendall (2015) obtuvieron resultados similares al estudiar a 23 familias que visitaban la exposición “Flip It, Fold It, Figure It Out! Playing with Math” en el Museum of Life and Science – Durham, EEUU (¡Dale la vuelta, dóblalo, descúbrelo! Jugando con Matemáticas – Museo de la Vida y la Ciencia). Según los autores, cuando los adultos recibieron instrucciones sobre el contenido de la exposición, se involucraron más en guiar a los niños, quienes, a su vez, emitieron respuestas más correctas sustentadas en razonamientos científicos, en comparación con los niños cuyos adultos no recibieron instrucciones. En este sentido, recordamos que los adultos no siempre dominan el contenido de la exposición (ejemplo 2), por lo que los paneles y rótulos pueden representar importantes recursos didácticos para ellos.

En el proceso de interacción con la exposición el código “interacción contemplativa” se produjo en 292 secciones. La expresividad del código se debe al carácter contemplativo de la exposición, ya que contenía materiales propios del trabajo correspondiente a un naturalista como algunos fósiles, ejemplares de plantas de herbario, animales vivos y taxidermizados, así como proyecciones, videos, fotos y mapas que dieron cuenta de la labor de Charles Darwin en el estudio y entendimiento del mundo natural. Los episodios que hacen referencia a este código se muestran en los ejemplos 4 a 6.

Ejemplo 4 (G2): V1: ¿Ya se murió Darwin? [observando la proyección sobre Darwin]./ V3: Ya [al finalizar la proyección]./ V1: Mira todo lo que tienen aquí, muy lindo e interesante.

Ejemplo 5 (G3): V1: ¡Mira esta! [apunta hacia el dibujo de una tortuga]./ V3: ¡Ay, ohhh!/ V1: Tiene la nariz igual que ella./ V3: Se parecen./ V1: Ohh [sorpresa] Mira este, tiene como pico!/ V3: [señala el camarón]./ V1: ¡Guau! ¿Ese es como ostra, camarón?/ V3: Como un langostino, un langostino./ V1: ¡Guau, a ver!

Ejemplo 6 (G7): V5: ¡Ah mira! ¡Está chiquitita!/ V1: No veo. No veo./ V2: ¿Está en el agua?/ V5: Si, por el calor. De aquí se ve. Mira de aquí, se ve la cabeza./ V1: Ahhh [sorpresa]./ V3: Pitón de la India./ V1: Es un Pitón de la India, papá./ V5: Vamos a ver aquel.

Al verificar las interacciones entre los visitantes y entre ellos y los objetos expuestos notamos que los animales taxidermizados atrajeron la atención de las familias. Ante ellos las interacciones se basaron en la identificación del animal a partir de su propio conocimiento como en ejemplo 5. En el ejemplo 6, la familia se posiciona frente al terrario y, a partir de la observación de la serpiente, propuso hipótesis para explicar el comportamiento del animal “V2: ¿Está en el agua?/ V5: Sí, por el calor”.

Nuestros resultados corroboran los de Allen (2002), cuyo estudio se llevó a cabo en la exposición “Frogs” (Ranas), ubicada en el museo de ciencias Exploratorium (San Francisco, EEUU), compuesta por objetos de manipulación y contemplación, como terrarios de ranas. Según el autor, las familias se posicionaron frente a estos animales y en su mayoría llevaron a cabo conversaciones de tipo perceptual – que resalta algo que llamó la atención y cubre solo la identificación y lectura de la etiqueta, y el tipo conceptual – no necesariamente interpretaciones complejas de lo que se muestra; así como cuando realizaban actividades que requerían el manejo de objetos. Sin embargo, los terrarios invitaban a las familias a “desacelerar” y observar con calma, reflexionar y formular hipótesis para explicar el comportamiento del animal. En nuestro caso la presencia de animales y proyecciones (ejemplo 4) se reflejó significativamente en las conversaciones, siendo un factor decisivo para que las familias dedicaran el 84% del tiempo de la visita a hablar sobre el objeto que contemplaba.

En este sentido, entender el papel que juegan los objetos en las visitas no guiadas es fundamental, ya que, en ausencia del mediador, las características físicas, biológicas y simbólicas engancharán al visitante, en función de sus conocimientos previos y también de la información que se muestre en los paneles y etiquetas (Achiam, May, Marandino, 2014). Así, al considerar que los objetos tienen la capacidad de conectar al visitante con la exposición y que los visitantes son libres de elegir qué contemplar, la multiplicidad de objetos combinada con las interacciones durante la visita puede inculcar en el visitante el deseo de aprender (Svabo, 2008).

Rice y Yenawine (2002) destacan la importancia de los objetos y otros elementos que compondrán la exposición al aclarar que cuando el diseño se centra únicamente en la explicación de hechos y conceptos, el visitante puede ponerse en una posición pasiva frente a la información y supongamos que se necesita un gran marco teórico para comprenderlo. En nuestro estudio no observamos visitantes pasivos, hecho que puede verse reforzado por la expresividad de este código y, en particular, el código de Conversaciones sobre temas de ciencia.

También en la interacción con la exposición el código “lectura de panel/texto/foto” correspondió al 32% del tiempo total de visitas (n = 248). Este resultado es un reflejo del diseño informativo y contemplativo de la exposición, promovido por el hecho de que las familias realizaron la visita sin la presencia de los mediadores. Los episodios que hacen referencia a este código se muestran en los ejemplos 7 y 8.

Ejemplo 7 (G10): V2: Un joven naturalista … Esta es la familia Darwin./ V1: ¿Cómo sabes?/ V2: Porque ahí dices [apunta para el panel].

Ejemplo 8 (G7): V1: Cuando lee que Darwin se casó con su prima: Papá, ¿con quién se casó? [pregunta después de leer o panel]./ V4: Bien, no sé, hay que leer./ V1: ¿Con su hermana?/ V4: Ah, no, su hermana no./ V1: Pero ahí dice: se casó con una mujer que ya conocía desde niño, a su prima-hermana Ema ¿Se casó con su prima? Ambas partes y sus familias acordaron que serían la pareja perfecta …/ V2: ¿Se casó con su prima-hermana?/ V4: Sí, es como si Marco se casase con Ana./ V1 y V2: Arghhh./ V4: O ustedes con Daniel./ V1 y V2: Arghhh./ V4: Es solo un ejemplo./ V1: Otro ejemplo, papá.

Al analizar las interacciones notamos que prevalecían dos tipos de lecturas: las rápidas y las largas. Las lecturas rápidas fueron tomadas por los niños y adolescentes y permitieron identificar o comprender el objeto expuesto como en el ejemplo 7. Las interacciones resultantes de estas lecturas permitieron aclarar dudas específicas sobre el objeto en contemplación por parte de la familia. Las lecturas largas, a su vez, se identificaron cuando los miembros de la familia leían en voz alta juntos y luego interpretan la información como en el ejemplo 8. En esto ejemplo, destacamos la interacción del grupo G7, en el que el padre logra explicar la relación entre Darwin y Ema tomando como ejemplo su propia familia, asociando la información con la realidad de los niños.

Los ejemplos muestran que el diseño informativo de la exposición contribuyó a las interacciones después de la lectura (Allen, 2002). Según Diamond (1986), la lectura de paneles de información y etiquetas de identificación ayuda a los adultos a comprender la información, que a su vez la transmite de forma simplificada, facilitando la comprensión de niños y adolescentes. Además, la lectura en voz alta y en conjunto también contribuye al conocimiento de los adultos y sirve para dar respuesta a las cuestiones planteadas durante la visita.

En este sentido, es importante resaltar la relevancia de los paneles de información y etiquetas de identificación para el aprendizaje de las familias estudiadas. El comportamiento observado en las familias de nuestro estudio es compatible con los estudios realizados en el contexto norteamericano según lo observado por Crowley et al. (2001) y Ellenbogen (2002), y, en el contexto latinoamericano, en Guimarães et al. (2019) y Cerqueira et al. (2016). Asimismo, la exploración conjunta de la familia, así como su implicación en las actividades de lectura e interpretación de información, se refleja en una mejor comprensión de los conceptos y fenómenos expuestos como consecuencia del rol asumido por los adultos, contribuyendo al desarrollo del pensamiento científico de los niños y los adolescentes (Fender, Crowley, 2007).

## ¿De qué hablaron las familias durante la visita?

En la dimensión “conversación”, el código “conversaciones sobre exposición” (funcionamiento, diseño, experiencia museística) fue el más marcado (n = 339) y estuvo presente en el 17% del tiempo total de visitas. Aunque las citas fueron de corta duración, su alta frecuencia se debe a la co-ocurrencia en las interacciones: visitante-visitante (n = 149) y visitante-módulo expositivo (n = 246). Los episodios que hacen referencia a este código se muestran en los ejemplos 9 a 10.

Ejemplo 9 (G6): V3: Mira su letra, mira las plantas que prensaba en los libros, las llevaba a Londres y las empacaban, pero antes de esto las clasificaban y las ponían esta planta es de tal lugar, tal hora, tal día. Y una vez que estaban en el barco, se ponían a estudiar y estudiar [explicando para V1 el montaje de las plantas de herbario].

Ejemplo 10 (G3): V3: Podrían poner unas luces acá, ¿no?/ V1: Aha. Podrían poner unas luces./ V3: Podrían poner unas luces y quedaría súper bonito, para ver bien. Porque no era tan grande, mira. A ver, 27 metros de largo [leyendo el panel]./ V1: Tan poquito. Tan chiquitito./ V3: En su cabina, compartida con otros dos hombres, tenía tres metros por 3,4 metros [lectura]./ V3: De aquí hasta aquí [madre usó el espacio de exhibición para demostrar las medidas del stand para su hija]./ V1: ¡Muy chiquitito!

Los ejemplos presentados muestran que las conversaciones sobre exposición (funcionamiento, diseño, experiencia de museo) ocurrieron cuando las familias buscaban explicar el funcionamiento de los objetos expuestos, hablaban sobre el diseño de la exposición, o incluso mientras observaban y señalaban algún objeto en la contemplación (ejemplos 9 y 10).

Callanan et al. (2020) estudiaron a 650 visitantes (familias con adultos y niños) durante una visita a la exposición de tres museos de ciencia infantil norteamericanos: Providence Children’s Museum (Museo de los Niños de Providence – Providence), Children’s Discovery Museum (Museo del Descubrimiento de los Niños – San José) y Thinkery (Pensamiento – Austin). Según este estudio las interacciones entre la familia conducen a una mayor proporción de conversaciones sobre la exposición, en comparación con las conversaciones no relacionadas con la exposición. Allen (2002) analizó el contenido conversacional de 98 visitantes (49 parejas: adulto-adulto y adulto-niño) que visitaron la exhibición “Frogs” (Ranas) y también informó que la proporción de conversaciones relacionadas con la exhibición fue mayor que en otras conversaciones. Asimismo, Guimarães et al. (2019), al analizar diez familias que visitan la exposición “Océanos” ubicada en el Museo de la Vida (Rio de Janeiro, Brasil), observaron que la mayoría de las conversaciones estaba relacionada con objetos y aparatos.

Cabe mencionar que las interacciones ejemplificadas en la Tabla 3 muestran que los adultos actuaron como facilitadores para la comprensión del objeto expuesto: ya sea para entender cómo se produce (ejemplo 9) o sus dimensiones (ejemplo 10), en estos casos adultos podrían haber llevado a cabo conversaciones científicas más profundas.

En cuanto al código temático de “conversaciones sobre ciencia”, encontramos que correspondía al 42% de interacciones entre familias (n = 307). El análisis de los vídeos mostró que este código co-ocurrió con otros tres códigos: visitante-visitante (n = 147), en general, después de la interacción contemplativa (n = 155), o después de la lectura del panel/texto/explicativo/foto (n = 102). Los episodios que hacen referencia a este código se muestran en los ejemplos 11 a 13.

Ejemplo 11 (G6): V3: Mirando el mapa con el camino de las embarcaciones: Se paró en las Islas Caimán. Luego en Cabo Verde, El Salvador, Río de Janeiro, las Islas Malvinas, también en Argentina que es la última parte de América Latina …, todo eso es Chile [apunta para el mapa]/ V1: Chi, Chi, Chile/ V3: Luego Galápagos, mira. Acá está México y mira de donde salió. Con el barco dando toda esta vuelta a América Latina, ¿por qué? Porque no hay paso. Hoy ya se puede atravesar por medio del canal de Panamá. Por aquí ahora pasan los barcos, por aquí y allá. Todo ese recorrido para llegar aquí, en el Ecuador, recuerda que el Ecuador divide la Tierra, es la mitad, ¿sí?/ V1: Sí!/ V3: Para arriba y para abajo, Hemisferio Norte y Hemisferio Sur. Estoy hablando, escúchame [hablando con V2]. Y en frente al Ecuador están las Islas Galápagos, donde se encuentran plantas, tortugas … Toda la vuelta al mundo en cinco años, increíble ¿no? En barco.

Ejemplo 12 (G1): V3: ¿Ya viste? ¿Ya viste que eso es una tortuga por dentro, hija?/ V1: Ese es un esqueleto … un gato, ¿ese es un gato? Es mejor un gato en la vida real. Cráneo de chiva. Esqueleto de rata. Esqueleto de rana toro, ¡rana toro! [risas] Esqueleto de conejo ¡Qué bonito! Uhhhh Mariposas. Uhhh un dragón como los dragones de China. ¡Uy, qué es esto! [lectura hecha por V1]./ V4: Un cráneo de un rinoceronte ¡Seguimos! Jajaja.

Ejemplo 13 (G3): V1: Esqueleto de rata, esqueleto de murciélago, esqueleto de tortuga, esqueleto de orangután, esqueleto de pez, ¡Guau! esqueleto de chiva, esqueleto de rata, ¡ahhhh! [sorpresa] esqueleto de jirafa [lectura], ¡si no lo había visto! Mira esta cosa que tienes aquí [señalando hacia un esqueleto]./ V3: ¿En la mitad de la cabeza?/ V1: Aham [incomprensible]./ V3: Todo eso es cartílago./ V2: ¿Todo eso?/ V3: Sí, desde aquí hasta acá [demuestra en su rostro] ¿Te acuerdas [refiriéndose al esqueleto humano] cuando te quitas un pedazo [mostrando la nariz], ¿cómo queda esta parte? se queda el hueso, para acá es cartílago./ V2: ¿Y esta parte dura es tipo cartílago?!/ V3: No. De aquí para atrás es el cráneo, de aquí para acá, cartílago [demuestra en el rostro de su hija].

Los ejemplos anteriores muestran que la lectura de datos, hechos y contenido científico disponible en los paneles de la exposición, así como la contemplación de los objetos expuestos fueron los elementos impulsores para que los adultos iniciaran conversaciones sobre temas de ciencia. En el ejemplo 11 es posible observar una asociación con conocimientos previos de adultos y niños. En el ejemplo 12, la identificación de los esqueletos y animales taxidermizados fue realizada por el niño a partir de la lectura de las etiquetas. En el ejemplo 13, las comparaciones, analogías y el conocimiento previo de los padres hicieron posible trasponer la información al esqueleto humano.

Si bien este código se dio de manera expresiva en el análisis, las conversaciones tuvieron poca profundidad sobre el conocimiento científico cubierto por la exposición. En todos los ejemplos los adultos se preocuparon por explicar los conceptos de forma simplificada. Atribuimos este resultado a la edad de los niños, especialmente en los grupos G1 (4 y 8 años) y G8 (3, 4 y 10 años), y posiblemente a la falta de dominio de conceptos por parte de adultos y/o niños y adolescentes, como se observa por Guimarães et al. (2019) en la exposición antes mencionada. Callanan et al. (2017), al estudiar a 83 familias que visitaban la exposición “Mammoth Discovery!” (¡Descubrimiento del Mamut!), ubicada en el Children’s Discovery Museum, reportaron que las conversaciones más profundas se correlacionaron con la edad del niño; sin embargo, su compromiso en brindar las explicaciones científicas se debió a las preguntas formuladas por los adultos, y no a las explicaciones dadas por ellos. En nuestro caso, el ejemplo 11 muestra la misma tendencia.

La presencia de esqueletos y animales taxidermizados llamó la atención de las familias y dio lugar a conversaciones que cubrieron solo la apreciación e identificación de características físicas (ejemplos 12 y 13). Allen (2002) y Cerqueira et al. (2016), al estudiar las conversaciones familiares en los museos de ciencia, también señalan que la mayoría de las conversaciones entre los visitantes fueron poco profundas y cubrieron aspectos perceptivos y conceptuales (como se dijo anteriormente).

En la misma dirección, Callanan, Jipson (2001) estudiaron las interacciones entre familias que visitan el Children’s Discovery Museum y reportaron que, entre las explicaciones dadas por los padres, el 54% son del tipo de conexiones causales – comprensión de un evento puntual, pero sin conectar con principios científicos abstractos. El 25% hace conexiones con experiencias previas, y el 12% son del tipo principios científicos: introducen conceptos y principios científicos abstractos. En nuestro estudio, no observamos explicaciones de principios científicos, las conexiones causales se pueden ver en el ejemplo 11, las conexiones con experiencias previas también fueron observadas por nosotros.

Las familias demuestran habilidades relacionadas con la ciencia, como observar los animales, hablar sobre su comportamiento, morfología, hacer preguntas, buscar respuestas, elaborar explicaciones basadas en el razonamiento científico y la colaboración conjunta que permitió la construcción de sentido. Sin embargo, es importante aclarar que en este artículo no profundizamos el análisis sobre conceptos evolutivos, que fue tema de un artículo específico, en el cual justamente analizamos las conversaciones sobre evolución y el tipo de razonamiento que utilizaron las familias para comprender y explicar los procesos evolutivos (Massarani et al., 2022).

Para concluir la dimensión “conversación”, el código Conversaciones en que se asocia a vivencias previas y vivencias personales tuvo una menor ocurrencia (n = 44), pero resultó ser importante para la construcción de sentido de las familias sobre los temas tratados. Las conversaciones se desarrollaron en situaciones donde los grupos relacionaban el contenido de la exposición con los conocimientos previos adquiridos a través de películas y viajes. Los episodios que hacen referencia a este código se muestran en los ejemplos 14 a 16.

Ejemplo 14 (G6): V1: Aquí. Aquí. Ven ver este [esqueleto] ahora. Oh, la cabeza de un kaiju. ¿Mamá, te acuerdas de Pacific River? [Película: Titanes del Pacífico]./ V3: Federico Solórzano Barreto./ V1: ¿Sí?/ V3: Bueno, ese es el nombre del museo de paleontología, no sabía que era Barreto./ V1: ¿Mami, recuerdas el del río Pacífico? ¿De los robots gigantes que luchan contra los extraterrestres? Mira, ¿no parece un kaiju, el alienígena?/ V3: ¿Pero de qué se trata?/ V1: Jirafa./ V3: Jirafa.

Ejemplo 15 (G3): V1: Mira ese que…/ V3: Ah, de las palomas, ¿te acuerdas que corriste de todas las palomas?/ V1: Eso me gusta mucho, la blanca y…/ V3: ¿La capuccino?/ V1: Aham. Esas dos están super lindas./ V3: Columba. Ese esta por todos lados, no?/ V1: Uhum./ V3: Este es silvestre. Está por todos lados, por la ciudad./ V1: ¿Está viva?/ V3: Ah, mira cómo está bonita, sus manos con plumas también./ V1: Qué bonitas.

Ejemplo 16 (G4): V2: Son mariposas, ¿ya viste? [hablando con su hijo]./ V1: Ay, se admira de unas mariposas, como las que yo agarro. Un día vi una de esas./ V2: ¿Si?/ V1: Ay, mariposas. Un día iba a querer una mariposa así lunática, pero sí más fuerte./ V2: ¿Viste?/ V1: Ya lo ví. Pero ese es el rey de las abejas y yo de las mariposas. Mira esas están chidas, pero más chidas están esas.

Al analizar los ejemplos anteriores se puede notar que las conversaciones fueron puntuales, corroborando el reducido tiempo de ocurrencia de este código (3% en relación al tiempo total de visita). Las conversaciones contaron con la participación activa de los niños que hicieron asociaciones con películas que disfrutaba la familia (ejemplo 14) y mostró aprecio por el animal contemplado (ejemplos 15 y 16). Para Crowley, Pierroux e Knoust (2014), al explorar conjuntamente la exposición, los adultos tienen la posibilidad de estimular el pensamiento científico de niños y adolescentes, motivándolos a reflexionar sobre lo que se está contemplando. Por tanto, como estrategia para mejorar la experiencia museística familiar y el aprendizaje de niños y adolescentes, los adultos conectan la exposición a experiencias previas para facilitar la comprensión de información y conceptos científicos (Allen, 2002).

El ejemplo 16 proporciona evidencia de esta estrategia en la que una pregunta elaborada por el padre le permitió al niño darle una respuesta inesperada y significativa. Callanan et al. (2017) también observaron que las preguntas abiertas (respuesta no implícita) elaboradas por los adultos llevaron a conversaciones más largas y a una mayor implicación de los niños con el tema expuesto, en comparación con preguntas cerradas (respuesta implícita) o conversaciones explicativas resultan en experiencias que son marcado en la memoria de los niños y adolescentes. Este es un carácter relevante de las exposiciones participativas, s cuando la información y los objetos expuestos se contextualizan a la realidad de los visitantes, la experiencia se traduce en un aprendizaje más profundo (Falk, Dierking, 2014), permitiendo a la familia experimentar la construcción colaborativa de los sentidos (Guimarães et al., 2019).

## ¿Qué emociones surgieron de las interacciones y conversaciones?

El código de “emoción” ocurrió en 199 tramos y correspondió al 7% del tiempo total de los vídeos analizados. Esto se debe a la naturaleza de este tipo de interacción, expresada en frases cortas, que abarcan una amplia gama de sentimientos. Los episodios que hacen referencia a este código se muestran en los ejemplos 17 a 19.

Ejemplo 17 (G9): V1: ¿Qué es eso? ¡Estás despierta, mira! una serpiente./ V4: Si, ¿está viva?/ V5: Si, está viva./ V1: ¡Qué miedo!.¡Me da miedo!/ V4: ¡No tocar!

Ejemplo 18 (G3): V1: Mira me fascina ese esqueleto. De caballo./ V3: Está padre./ V1: ¡Está padrísimo!

Ejemplo 19 (G5): V1: ¡Mira el chango! Es de mentira… Pobrecito, el chango. Pobrecito. Sí, los ojos se ven muy reales./ V2: Sí [observando un macaco taxidermizado].

Muchas fueron las respuestas emocionales (positivas y negativas) de los visitantes al ver los animales vivos o taxidermizados, como miedo (ejemplo 17), lástima y disgusto (ejemplo 19), encantamiento y alegría (ejemplo 18). Aunque las emociones fueron puntuales, fueron cruciales para la implicación inicial de las familias con la exposición, y este interés es fundamental para que se produzca el aprendizaje (NRC, 2009).

Falk y Gillespie (2009) estudiaron a 22 visitantes de la exposición temporal “Goose bumps: the science of fear” (Piel de Gallina: la ciencia del miedo) en un centro de ciencias en los Estados Unidos y a 15 visitantes que visitaron la exposición permanente en el mismo centro de ciencias. Observaron que el diseño pensado para emocionar al visitante de la exposición temporal generó un mayor entusiasmo e involucramiento en los visitantes, quienes, a su vez, presentaron mejores resultados de aprendizaje en comparación con los visitantes que únicamente conocieron la exposición permanente.

Asimismo, Guimarães et al. (2019) observaron que la exposición “Océanos” provocó un amplio abanico de sentimientos en las familias, pero los de carácter afectivo destacaron en el módulo que abordó el tema de la basura en los océanos. Según los autores el módulo en cuestión permitió a las familias reflexionar sobre sus propios hábitos y valores en cuanto a la disposición de la basura, demostrando que la exposición alcanzó su objetivo: sensibilizar a los visitantes sobre el impacto de la basura en los océanos.

Dado lo anterior, es evidente que el diseño de la exposición permitió a los visitantes vivir una experiencia multifacética al interactuar con los objetos y entre ellos, provocando una respuesta emocional que generó entusiasmo. Según Falk y Gillespie (2009), el entusiasmo y la valencia actúan directamente sobre el aprendizaje por su influencia en la memoria e indirectamente porque están relacionados con la atención. Así, aunque las emociones observadas por nosotros no siempre han sido positivas, es posible ver que las experiencias vividas han contribuido al aprendizaje de las familias.

## Consideraciones finales

La visita al Museo Trompo Mágico brindó la vivencia de situaciones que despertaron el interés y la emoción de los grupos familiares, características esenciales para el aprendizaje colaborativo. En cuanto a las interacciones, ciertamente son reflejos del diseño informativo y contemplativo de la exposición y se dieron entre dos actores que conforman el “triángulo pedagógico”: los módulos expositivos y los propios visitantes.

Al analizar el contenido de las conversaciones se evidencia que las familias se mantuvieron enfocadas y compartieron conocimientos sobre el tema abordado por la exposición durante toda la visita. Al considerar el papel que jugaron los adultos en este proceso, se destaca su importancia en la decodificación de información y conceptos científicos. Igualmente importantes fueron los paneles de información y las etiquetas que, a través de la lectura, proporcionaron soporte conceptual para las interacciones. También es interesante notar que, si bien la participación de los adultos se dio en diferentes intensidades entre las familias estudiadas y que las conversaciones sobre temas de ciencia no fueron muy profundas, buscaron facilitar la comprensión por parte de niños y adolescentes estableciendo conexiones entre información y conceptos de experiencias previas, así como a través de comparaciones y analogías entre objetos contemplados.

En este sentido la autonomía de las familias para recorrer la exposición con libertad y guiados por sus propios intereses fue el detonante del aprendizaje por libre elección, validando el hecho de que la forma en que ocurrió se debió al contexto de los visitantes y al de la exposición, facilitado por conexiones cognitivas y emocionales. Así, los visitantes pudieron involucrarse con temas socialmente significativos con la posibilidad de aplicar los conocimientos adquiridos durante la visita a su vida cotidiana.

Finalmente, el protocolo utilizado para el análisis de interacciones y conversaciones permitió una mejor comprensión de las características que aportan el aprendizaje y fomentan el interés de las familias por las materias científicas. Los resultados obtenidos pueden orientar a los profesionales de museos y educadores a diseñar exposiciones con diferentes características físicas y simbólicas que favorezcan conversaciones e interacciones con la ciencia. Por ejemplo, en el caso de la exposición estudiada, observamos que los objetos que más llamaron la atención de las familias fueron los animales taxidermizados acompañados de la lectura de paneles/textos/fotos explicativos, ya que permitieron la participación activa de las familias, el establecimiento de conexiones entre la información científica con sus experiencias previas y la creación conjunta de sentido. De esta manera consideramos haber avanzado en la comprensión del aprendizaje desde la perspectiva sociocultural en los museos en el contexto latinoamericano.
